# Lifelong Evolution of Autoreactive Plasma Cell Numbers, Affinity, and Anatomic Location in Arthritic K/BxN Mice

**DOI:** 10.1002/art.43436

**Published:** 2026-02-17

**Authors:** Thibault Vanhoucke, Carlos Castrillon, Odile Richard‐Le Goff, Angga Perima, Matteo Broketa, Andrew D. Griffiths, Patrick England, François Huetz, Pierre Bruhns

**Affiliations:** ^1^ Institut Pasteur, Université de Paris, Unit of Antibodies in Therapy and Pathology, Inserm UMR1222 75015 Paris France; ^2^ Sorbonne Université, Collège Doctoral Paris France; ^3^ Laboratoire de Biochimie, ESPCI Paris, PSL Research University, CNRS UMR8231 Chimie Biologie Innovation Paris France; ^4^ Institut Pasteur Université Paris Cité, CNRS UMR3528, Plateforme de Biophysique Moléculaire Paris 75015 France

## Abstract

**Objective:**

The spontaneous K/BxN mouse model of rheumatoid arthritis has been used extensively to study chronic inflammation, contribution of immune cells, and the primordial role of autoreactive antibodies in disease initiation and severity. Only the ubiquitous enzyme glucose‐6‐phosphate isomerase (GPI) is the target of IgG autoantibodies secreted by autoreactive plasma cells and plasmablasts in K/BxN mice. Strikingly, the appearance and evolution of these autoreactive IgG‐secreting cells (IgG‐SC) remain unstudied.

**Methods:**

Here, we quantitatively and qualitatively investigated the plasmablast and plasma cell responses by measuring the affinity of their secreted antibody for GPI from single cells in cohorts of K/BxN mice from 3 weeks to 87 weeks old.

**Results:**

Analysis of more than 36,000 individual IgG‐SC from spleen, popliteal lymph nodes, and bone marrow revealed high intercellular variability in affinity for GPI, with variations over three logs, with stable secretion rates over the life of the mice. Autoreactive IgG‐SC were detectable at 3 to 4 weeks and reached peak proportions of IgG‐SC at 35 weeks before stabilizing. High‐affinity anti‐GPI IgG‐SC appeared only transiently in the 6 to 9 weeks postnatal window, whereas low‐affinity IgG‐SC represented more than 80% of the response at all time points. Serum anti‐GPI IgG antibodies evolved in a similar kinetic manner, peaking in proportion to total IgG and in affinity for GPI at 35 weeks.

**Conclusion:**

Our results report the dynamic nature of the autoimmune B cell response in the K/BxN model, revealing a transient phase of antibody affinity maturation early in disease that allows high‐affinity antibody responses.

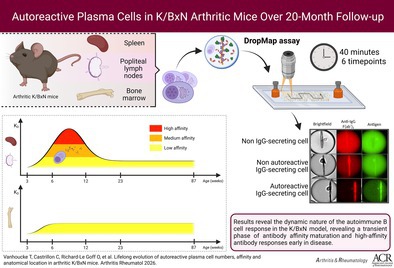

## Graphical abstract

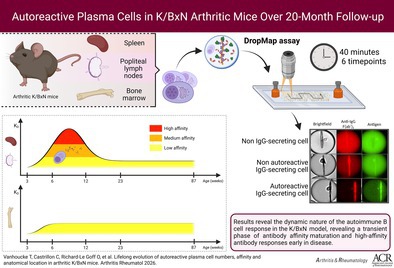

## INTRODUCTION

The hallmark antibodies in rheumatoid arthritis (RA) are rheumatoid factors and antibodies to posttranslationally modified proteins, for example, citrullinated protein, detectable in 60% to 70% of patients with RA but also antibodies to ubiquitously expressed enzymes like glucose‐6‐phosphate isomerase (GPI) antibodies in subgroups of patients with RA.^1^ The presence of autoantibodies in patients correlates with more severe disease course.^2^ Targeting the source of antibody production, using anti‐CD20 rituximab to deplete B cells^3^ or using anti‐CD38 daratumumab to deplete plasma cells,^4^ has proven efficacious in RA.

To understand the role of autoantibodies in RA, spontaneous mouse models of RA have been developed, such as the K/BxN model.^5^ In this model, arthritis arises spontaneously by the production of autoantibodies targeting GPI that deposits on the articular cartilage. K/BxN mice are C57BL/6 × nonobese diabetic (NOD) F1 mice, which express the KRN transgenic T cell receptor (TCR) that recognizes a peptide from GPI presented by the NOD‐encoded I‐Ag^7^ major histocompatibility complex class II molecule. This leads to a massive T cell and concomitant B cell activation, with high quantities of anti‐GPI autoantibodies produced, causing cartilage destruction and symptoms resembling RA. Pathogenic IgG anti‐GPI autoantibodies appear by three weeks old,^6^ accumulating at a high concentration in K/BxN serum (>6 mg/mL).^7^

This massive adaptive immune response against GPI in K/BxN mice relies on KRN TCR‐expressing T cell subsets, for example, Th17 cells, T follicular helper (Tfh) cells, and dendritic cells that promote extensive polyclonal B cell activation during germinal center (GC) reactions that start in the lymph nodes draining the distal joints.^8^, ^9^, ^10^ Both T cell–independent activation of marginal zone B cells by circulating GPI and T cell–dependent activation of GC B cells by antigen‐presenting cells contribute to the pool of anti‐GPI short‐lived plasmablasts and long‐lived plasma cells.^11^ Initial descriptions of IgG‐secreting cells (IgG‐SC), namely, plasmablasts and plasma cells, in 8‐week‐old K/BxN mice suggested their very high proportion in lymph nodes and spleen but lower proportions in blood and bone marrow. Anti‐CD20 B cell depletion experiments reduced their numbers 10‐fold in lymph nodes and spleen, and 100‐fold the circulating anti‐GPI IgG titers, suggestive that CD20^+^ short‐lived plasmablasts are largely predominant over CD20^−^ long‐lived plasma cells in the K/BxN model.^12^ The dynamics of IgG‐SC generation and anatomic distribution have, however, never been described in this extensively used mouse model of RA. The model has been considered to raise low‐affinity anti‐GPI antibody responses due to the strong extrafollicular B cell responses and short‐lived plasma cell responses, but subnanomolar affinity monoclonal antibodies (mAbs) could be isolated from K/BxN mice that were approximately 8 weeks old,^6^ indicating the generation of high‐affinity anti‐GPI B cells in this autoimmune model of RA.

Herein, we investigated over 20 months the dynamics of IgG‐SC generation and their anatomic distribution among the popliteal lymph nodes (PLN), spleen, and bone marrow of K/BxN mice. Our data identify a narrow window of time for autoreactive B cell expansion and affinity maturation, restricting affinity enhancement to one log on average over the lifetime of K/BxN mice.

## MATERIAL AND METHODS

### Reagents

Mouse GPI (CSB‐EP009717MO) was purchased from Cusabio. GPI was fluorescently labeled using AlexaFluor488 (AF488) NHS‐Ester dye (46403; Thermo Fisher Scientific). Anti‐GPI mAbs clones B10.8, D24.9, D24.4, and E4.6 were produced from hybridomas generated in house and purified by affinity chromatography using an ӒKTA pure fast protein liquid chromatography instrument on a HiTrap Protein G column and desalted on a HiTrap desalting column (all Cytiva).

### Mice breeding

K/BxN and BxN mice were bred at Institut Pasteur by crossing NOD (purchased from Charles River Laboratories) mice with transgenic KRN mice or C57BL/6 wild‐type mice, respectively. Wild‐type BALB/c mice were purchased from Janvier Labs. All animal care and experimentation was conducted in compliance with the guidelines and specific approval of the Committee for Ethics in Animal Experimentation (Institut Pasteur) and by the French Ministry of Research.

### Aqueous phase I: cell preparation for droplet encapsulation

Cells from spleen, PLN, and bone marrow from tibia and femur were collected and filtered through a 100‐μm cell strainer before red blood cell lysis (buffer 555899; BD Biosciences). Cell density was adjusted at 30 × 10^6^/mL in DropMap buffer (RPMI without phenol red [Gibco], 0.1% Pluronic F68 [Gibco], 25 mM HEPES pH 7.4 [Gibco], 5% KnockOut serum replacement [Thermo Fisher Scientific], and 0.5% human serum albumin [Sigma‐Aldrich]) to achieve an average number of approximately 0.3 cell per droplet. For the generation of data points for the calibration curves, anti‐GPI mAbs were diluted in DropMap buffer.

### Aqueous phase II: beads and reagents preparation

Streptavidin‐coated paramagnetic nanobeads (03231; Ademtech) were incubated in Dulbecco's phosphate‐buffered saline (DPBS) without calcium and magnesium (Gibco) containing 1 μM CaptureSelect biotin anti‐κ light chain conjugate (Thermo Fisher Scientific) for 20 minutes at room temperature and washed and incubated in 5% Pluronic F68 solution (P6866; Thermo Fisher Scientific) for 20 minutes at room temperature. Nanobeads were incubated in DropMap buffer for 20 minutes at room temperature, washed in DPBS and resuspended in DropMap buffer containing 60 nM of AF488‐labeled GPI (final in‐droplet concentration 30 nM) and 150 nM of an AF647‐labeled rabbit anti‐mouse IgG F(ab’)_2_ (final in‐droplet concentration: 75 nM; 315‐606‐046; Jackson ImmunoResearch).

### DropMap: material production, droplet encapsulation, and data acquisition

The silicon wafer, the chip with the droplet chamber, and the droplets were produced, as we described previously.^13^ Images of the droplet chamber were acquired on an inverted microscope with a motorized stage (Ti2‐Eclipse, Nikon) and a high‐speed camera (Orca Flash 4.0, Hamamatsu) at room temperature every 7.5 minutes over a period of 37.5 minutes, as reported.^13^

### Image analysis and calculation

**Figure 1:**
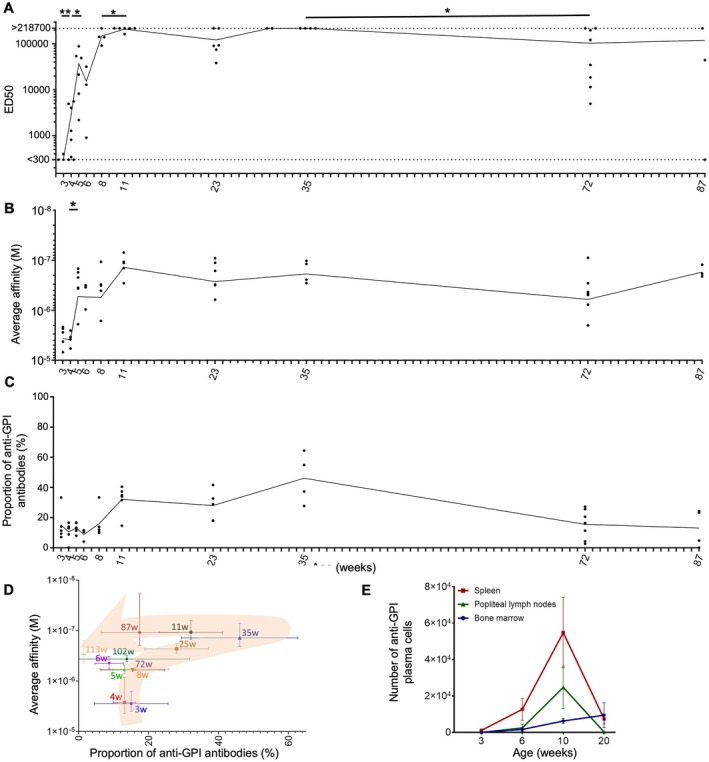
Dynamics of circulating anti‐GPI antibodies in K/BxN. (A) ED50 computed from anti‐GPI enzyme‐linked immunosorbent assay on serum taken at indicated ages from K/BxN mice. Values outside of dilution range were indicated as either <300 or >218,700. Surface plasmon resonance measures of average anti‐GPI affinity (B) and proportion of anti‐GPI IgG autoantibodies (C) among circulating IgG on serum taken at indicated ages from K/BxN mice. (D) Kinetic evolution of average anti‐GPI affinity and autoantibody proportion among circulating IgG, with mean ± SEM indicated. The orange arrow indicates the trajectory taken for average affinity and autoantibody proportion over time from 3 weeks (start of line) to 113 weeks (arrowhead). (E) Kinetic evolution of the number of anti‐GPI B220^+^ CD38^+^ plasma cells and/or plasmablasts in indicated tissues. (A–C) Consecutive timepoints were compared using a Mann‐Whitney test. *, *P* < 0.05; **, *P* < 0.01. ED50, median effective dose; GPI, glucose‐6‐phosphate isomerase.

Images were analyzed using a custom‐made MATLAB script (MathWorks) to identify each droplet and its beadline. In each fluorescence channel and for each droplet, the pixel intensities of the beadline and the mean pixel intensities except the beadline (background fluorescence) were extracted. Droplet fluorescence relocation at each time point was calculated by dividing the intensity of the beadline by that of the background. Droplets showing between the first and last time point a minimum relocation >1.2, a net difference >0.025, and a minimal slope of 0.004 were considered positive for IgG secretion. These droplets were visually assessed for the presence of a single cell or two cells (droplets with more than two cells were excluded), no droplet movement between image acquisitions, and the absence of fluorescence artifacts, as described.^13^ The computed metrics of AF647 and AF488 fluorescence relocation over time were converted into IgG secretion rates and affinity for GPI by using calibration curves generated with a panel of mAb of known affinities for GPI encapsulated in droplets at different concentration (Figure 1C; Supplemental Figure 1B).

### Anti‐GPI enzyme‐linked immunosorbent assay

The 96‐well plates (Costar) were coated with 5 μg/mL of rabbit GPI (P9544; Sigma Aldrich) in carbonate/bicarbonate buffer (pH 9.6), blocked with phosphate‐buffered saline (PBS) with Tween containing 2% bovine serum albumin (BSA; Roche), washed and incubated with K/BxN or BxN mice serum samples diluted 1:300 to 1:218,700. Bound antibodies were detected by addition of goat anti‐mouse IgG Fc fragment horseradish peroxidase conjugated (A90‐131Ps; Bethyl Laboratories) at 1:4,000, and revealed using o‐phenylenediamine dihydrochloride substrate (P9187; Sigma‐Aldrich), stopped by addition of 2 M sulfuric acid. Absorbance was recorded at 492 nm and 620 nm.

### Kinetics measurement by surface plasma resonance

For surface plasmon resonance (SPR) analysis (Biacore, Cytiva), rabbit anti‐mouse IgG Fcγ fragment‐specific antibody (315‐005‐046; Jackson ImmunoResearch) were immobilized on Sensor CM5 chips (Cytiva). IgG contained in K/BxN or BxN serum were captured during 300‐second injections. Mouse GPI was injected at various concentrations (2,000 nM, 667 nM, 222 nM, 74 nM, and 25 nM) in 1× PBS plus 0.01% (volume/volume) Tween 20 plus 0.2 mg/mL BSA for 300 seconds. Flow paths were regenerated by two cycles of 10 mM glycine hydrogen chloride (Glycine‐HCl pH 5) and one cycle of 10 mM sodium hydroxide. Polyclonal affinity for mouse GPI and maximal response when all ligand is occupied (Rmax) was determined using 1:1 “steady state affinity” modeling (Biacore T200 Evaluation software 3.1, Cytiva). Percentage of anti‐GPI IgG antibodies was calculated with the following formula (M_GPI_, molar mass of GPI; M_IgG_, molar mass of IgG):

$$\%=\frac{R_{\max}}{\mathrm{IgG}\;\text{capture level}\times \frac{M_{\mathrm{GPI}}}{M_{\mathrm{IgG}}}}\times 100$$

### Flow cytometry

To identify GPI–specific plasma cells and plasmablast by flow cytometry, single‐cell suspensions from spleen, PLN, and bone marrow were permeabilized and stained for B220‐, CD138‐, and AF488‐labeled GPI. Control experiments used AF488‐labeled ovalbumin instead of GPI and demonstrated no positive plasma cells. Flow cytometry acquisition was performed on a MacsQuant cytometer (Miltenyi Biotec) and analyzed on FlowJo software (BD Biosciences).

### K/BxN‐passive arthritis (serum transfer experiment)

K/BxN serum was collected from the same mice that were used for DropMap experiments, the day of the sacrifice. One pool of serum was generated using serum from K/BxN mice aged 6 to 12 weeks old and another from serum collected from K/BxN mice aged 72 to 83 weeks old. As a negative control, a pool of serum was generated using serum from KRN transgenic C57BL/6 mice aged 8 to 14 weeks old. Wild‐type BALB/c mice (purchased from Janvier Labs) were injected intraperitoneally with 200 μL serum on day 1 and 3. Ankle swelling was evaluated by ankle thickness measured separately on the right and left posterior paw using a caliper (Kroeplin S0247) from day 1 to day 12.

### Statistics

GraphPad Prism version 10.4.1 was used for all analyses. Data were not normalized or normalized before being analyzed. All data points present single‐cell values unless otherwise stated. Samples were not pooled unless explicitly stated. Affinity and secretion data were determined to be neither normally distributed nor log normally distributed according to the D'Agostino and Pearson, Anderson‐Darling, and Kolmogorov‐Smirnov tests. Paired or unpaired *t*‐tests and Mann‐Whitney tests were used for comparing samples or groups of samples. Kruskal‐Wallis test with Dunn's test for multiple comparisons was used on age‐grouped affinity values from DropMap data in Supplemental Figure 2F. Two‐way analysis of variance with multiple comparison was used to determine significance for data from K/BxN‐passive arthritis experiments. Statistical significance was set to a *P* value of ≤0.05.

### Data and materials availability

Further information and requests for resources, reagents, and material should be directed to and will be fulfilled by the lead contact, Pierre Bruhns.

## RESULTS

K/BxN mice develop arthritic symptoms from approximately 4 weeks old and survive up to 85 to 120 weeks old. Anti‐GPI autoantibodies were detectable starting at 3 weeks old, increased rapidly until 10 to 11 weeks old, and plateaued until 35 weeks old before modestly but significantly reducing afterwards (Figure 1A). We evaluated the evolution of this circulating anti‐GPI antibody pool in terms of the specific fraction prevalence and affinity for GPI by SPR measures on K/BxN mouse serum for 14 time points from week 3 to week 87. Serum anti‐GPI IgG polyclonal affinities evolved from a very poor 4 μM at 3 weeks old to their peak at 120 nM at week 11 and remained relatively stable until 87 weeks old (Figure 1B). The proportion of anti‐GPI IgG autoantibodies among all IgG evolved in successive plateaus from 10% to 15% between week 3 and week 8, to 30% to 46% between week 11 and week 35, before reducing back to 15% between week 72 and week 87 (Figure 1C). These data highlight three different phases of anti‐GPI IgG in circulation and presumable B cell responses in parallel with the following: (1) one log improvement of affinity for GPI (weeks 3–8); (2) increase in anti‐GPI IgG proportion with 0.5 log affinity improvement (weeks 10–35); and (3) reduction in anti‐GPI IgG proportion with minimal variations in affinity (>35 weeks). Analyzing rare samples of >100‐week‐old K/BxN mice confirmed this evolution with anti‐GPI IgG proportion down to 1% at 113 weeks without significant loss in affinity compared with 35‐week‐old mice (Figure 1D; a compilation of Figures 1B and 1C). Altogether, these data suggest early life appearance of anti‐GPI IgG^+^ plasmablasts and plasma cells that contribute to the circulating pool of anti‐GPI IgG, with a rapid increase in cell numbers and affinity maturation of the B cell response up to approximately 10 weeks old, followed by a slower evolution until approximately 35 weeks old.

To confirm this hypothesis on autoreactive plasma cell dynamics in the first weeks of life of K/BxN mice, we analyzed plasmablasts and plasma cells (defined as B220^lo/−^CD138^hi^) and among them GPI‐specific plasma cells by flow cytometry on permeabilized cells from spleen, PLN, and bone marrow of K/BxN mice between 3 and 20 weeks old. GPI‐specific and nonspecific plasmablast and plasma cell numbers increased rapidly in spleen and PLN until week 10, before decreasing by week 20 (Figure 1E; Supplemental Figure 1A and 1B). In the bone marrow, numbers and abundance of GPI‐specific plasma cells remained lower but steadily increased up to week 20 (Supplemental Figure 1A and 1B). To further understand what factors may drive these dynamics, we characterized Tfh cells (defined as B220^−^ CD4^+^ PD‐1^hi^ CXCR5^hi^) and GC B cells (defined as B220^+^CD4^−^GL7^+^FAS^+^) numbers and proportions by flow cytometry in the same timeframe. Tfh cell numbers and proportions among total T cells increased in spleen from 3 to 20 weeks, whereas in PLN they decreased after 10 weeks (Supplemental Figure 1C). In both organs, ≥85% of these Tfh cells expressed the KRN TCR (vβ6), suggesting their involvement in anti‐GPI autoreactive reactions (Supplemental Figure 1D). GC B cell numbers and proportions among total B cells also increased rapidly until week 10, before reducing or plateauing (Supplemental Figure 1E). The combined reduction of GPI‐specific Tfh cells and GC B cells in spleen and PLN after 10 weeks old may be responsible for the decrease in GPI‐specific plasma cells and plasmablasts after week 10 and may also contribute to the lack of anti‐GPI IgG affinity maturation after week 10 observed in polyclonal serum of K/BxN mice (Figure 1B).

**Figure 2:**
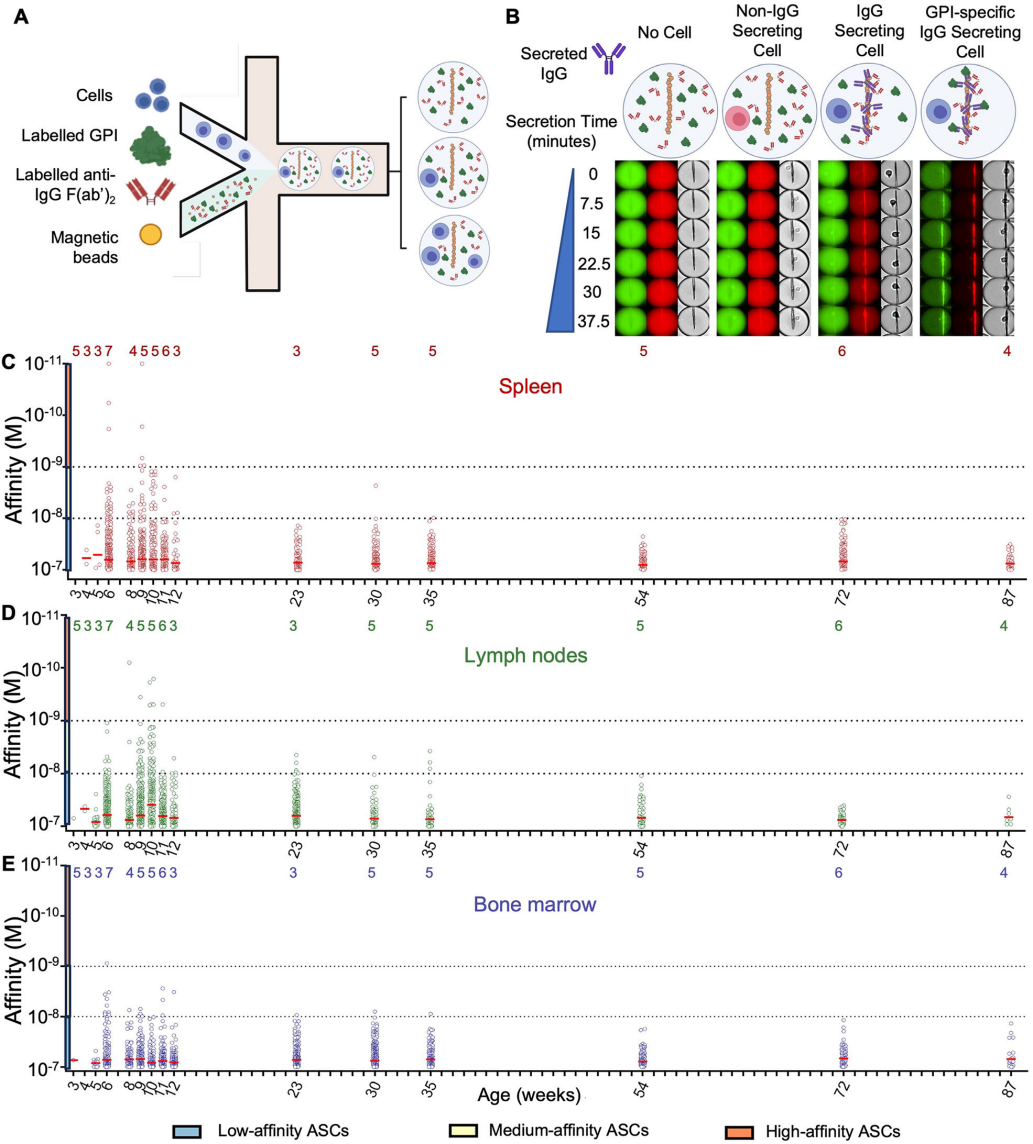
Kinetic evolution of IgG antibody‐secreting cell affinity for GPI in spleen, popliteal lymph nodes, and bone marrow. (A) Schematic showing the inputs and outputs of microfluidic encapsulation. Cells (top) are flowed in parallel with bioassay components [GPI Alexa Fluor 488, magnetic beads coated with anti‐κ VHH, and anti‐IgG F(ab′)_2_ Alexa Fluor 647] on the droplet nozzle. Oil flow closes the collected aqueous phases (cell component and bioassay component) into a water‐in‐oil droplet. The column of droplets depicts the potential outcomes of cell encapsulation. (B) Schematic showing the composition of the DropMap assay and potential changes to the assay as it progresses. In the absence of a cell (outer left) or with a non–IgG secreting cells (inner left), there is no change in GPI (green) or anti‐IgG F(ab′)_2_ (red) fluorescence distributions over time. A non‐GPI–specific IgG secreting cell (inner right) in the droplet causes anti‐IgG F(ab′)_2_ fluorescence to relocate to the beadline over time. A GPI‐specific IgG secreting cell (outer right) causes both GPI and anti‐IgG F(ab′)_2_ fluorescence to relocate to the beadline. IgG‐SC harvested from the spleen **(C)**, the popliteal lymph nodes, (D) and bone marrow (E) from 3‐week‐old to 87‐week‐old mice and characterized using DropMap for anti‐GPI affinity. Horizontal red lines represent the mean affinity for each timepoint. Affinity values better than 10 pM were plotted at 10 pM. The number of independent DropMap experiments for each timepoint is indicated on top of the graph. Colored boxes next to the y‐axis indicate low (blue), medium (yellow), and high (orange) affinity, separated by dotted horizontal lines. ASC, antibody‐secreting cell; GPI, glucose‐6‐phosphate isomerase. Color figure can be viewed in the online issue, which is available at http://onlinelibrary.wiley.com/doi/10.1002/art.43436/abstract.

We sought to analyze the affinity maturation of the anti‐GPI response in K/BxN mice by measuring over time the affinity for GPI of IgG secreted by IgG^+^ plasmablasts and plasma cells, which here we collectively term IgG‐SC. We thus adapted the single‐cell bioassay in microfluidic droplets termed “DropMap” that we have described previously,^13^ allowing a direct characterization of GPI‐specific IgG‐SC in terms of numbers, IgG secretion, and affinity of their IgG for GPI. Cells from spleen, PLN, and bone marrow of K/BxN mice were coencapsulated with paramagnetic nanobeads and fluorescent bioassay reagents in droplets (Figure 2A) before being immobilized within an observation chamber and imaged over 37.5 minutes by time‐lapse fluorescence microscopy (Supplemental Figure 2A). The nanobeads are precoated with an anti‐mouse kappa κ light chain nanobody to capture secreted Igs onto the beads that align into a stick‐like shape (termed beadline) under a magnetic field. This beadline acts as a physical surface for a fluorescent sandwich immunoassay revealing IgG,κ secretion by the cell inside the droplet and binding of that IgG,κ to GPI (Figure 2B; Supplemental Figure 2B). Image analyses extract binding metrics that are converted using reference curves (Supplemental Figure 2C and 2D) into IgG secretion rates within a 4 to 800 molecules/s range and into affinity (K_D_) for GPI within a 2.3 to 100 nM range. Therefore, hereafter IgG‐SC–secreting IgG interacting with GPI at a computed K_D_ < 100 nM are considered GPI specific, with K_D_ values <2.3 nM mathematically extrapolated.

In spleen and PLN, anti‐GPI IgG‐SC were detected from 3 and 4 weeks, respectively, to 87 weeks with affinities for GPI ranging from a few picomolar to 100 nM (mean affinity approximately 65 nM). Low‐affinity IgG‐SC (K_D_ ≥ 10 nM) predominated whereas high‐affinity IgG‐SC (K_D_ < 1 nM) could only be detected between 6 and 9 weeks old or 8 and 11 weeks old in spleen and PLN, respectively (Figure 2C and 2D). In the bone marrow, anti‐GPI IgG‐SC were detected starting 3 weeks old with affinities for GPI ranging from 0.86 to 100 nM (mean affinity approximately 75 nM). Only low‐affinity IgG‐SC, but no high‐affinity IgG‐SC (except one at week 6), were detected in the bone marrow (Figure 2E). Overall, anti‐GPI IgG‐SC appeared in spleen, PLN, and bone marrow of K/BxN mice immediately before the onset of disease symptoms and rapidly increased in numbers until a peak at around week 10 before decreasing to a plateau. The mean anti‐GPI affinity of IgG‐SC in all three compartments did not vary significantly from one time point to another over the lifetime of the mice (Supplemental Figure 2E), with all time points >80% low‐affinity IgG‐SC and ≤2.3% high‐affinity IgG‐SC, the latter cells detectable only during a narrow timeframe, from 6 to 11 weeks (Supplemental Figure 3). However, when compared with other age groups, anti‐GPI IgG‐SC from the 6‐week to 12‐week age group demonstrated significantly higher affinities than those from older age groups (Supplemental Figure 2F), which is suggestive of a temporal decline in affinities among anti‐GPI IgG‐SCs in the PLN and spleen and, to a lesser extent, in the bone marrow.

**Figure 3:**
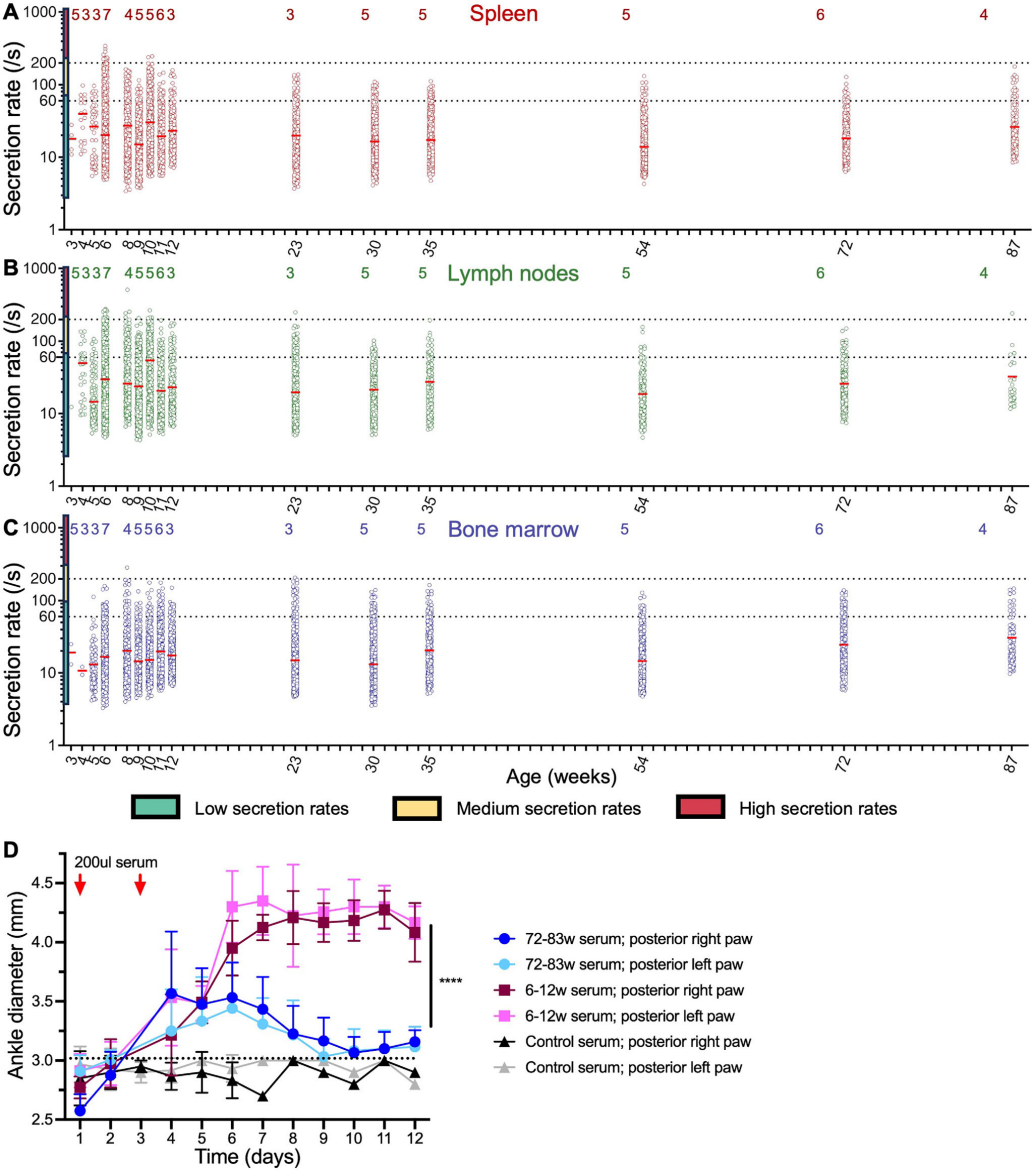
Kinetic evolution of IgG secretion from IgG‐secreting cells in spleen, popliteal lymph nodes and bone marrow, and evolution of proarthritic properties of K/BxN serum. IgG secreting cells harvested from the spleen (A), the popliteal lymph nodes (B), and bone marrow (C) from 3‐week‐old to 87‐week‐old mice and characterized using DropMap for IgG secretion (IgG molecules/s). Horizontal red lines represent the mean secretion rate for each timepoint. The number of independent DropMap experiments for each timepoint is indicated on top of the graph. Colored boxes next to the y‐axis indicate low (blue), medium (yellow), and high (orange) secretion rates, separated by dotted horizontal lines. (D) K/BxN serum transfer experiment in wild‐type BALB/c mice using serum collected from 6‐week‐old to 12‐week‐old or 72‐week‐old to 83‐week‐old K/BxN mice (n = 6) or control KRN mice (n = 3). The “6‐12w serum” curves were compared two‐by‐two with “72‐83w serum” curves using a two‐way analysis of variance multiple comparison test. ****, *P* < 0.0001. Color figure can be viewed in the online issue, which is available at http://onlinelibrary.wiley.com/doi/10.1002/art.43436/abstract.

The poor affinities of IgG secreted by IgG‐SC for GPI might be compensated by the large fraction of anti‐GPI IgG‐SC among all IgG‐SC that reached >20% on average with peaks at approximately 50% (Supplemental Figure 4). By quantifying, using DropMap, the secretion rate of IgG by every IgG‐SC analyzed in this study, we measured secretion rates ranging from 3 IgG/s to 336 IgG/s (mean 33 IgG/s) in spleen, from 4 IgG/s to 510 IgG/s (mean 33 IgG/s) in PLN, and from 3 IgG/s to 284 IgG/s (mean 21 IgG/s) in bone marrow (Figure 3A–C). Peak secretion rates were measured between 6 and 11 weeks old with outliers above 200 IgG/s representing <3% of all IgG‐SC (Supplemental Figure 5). No correlation between secretion rates and affinity for GPI was identified (Supplemental Figure 6A–C), with non‐GPI–specific IgG‐SC measured at higher IgG secretion rates than GPI‐specific IgG‐SC in PLN and bone marrow but not significantly in spleen (Supplemental Figure 6D–F). The increase in anti‐GPI IgG content over time in circulation is therefore not attributable to enhanced IgG secretion but uniquely to the increasing fraction and maturation of autoreactive IgG‐SC.

In order to determine whether the 6‐week to 11‐week period in the life of K/BxN mice demonstrating the presence of high‐affinity anti‐GPI IgG‐SC leads to the formation of a circulating a pool of antibodies with higher proarthritic properties than a period without these high‐affinity anti‐GPI IgG‐SC, we collected and pooled serum from K/BxN mice aged 6 to 12 weeks old and 72 to 83 weeks old, as well as from control KRN mice. As expected, K/BxN serum transfer, but not KRN serum transfer, led to clinical signs of inflammatory RA, as measured by increased ankle thickness. Notably, serum from K/BxN mice aged 6 to 12 weeks old induced significantly more severe and prolonged ankle swelling than serum from K/BxN mice aged 72 to 83 weeks old (see Figure 3D).

## DISCUSSION

Our study describes the dynamics of IgG‐SC over 20 months in K/BxN mice, starting one week before disease onset. It identifies a six‐week time window that supports rapid anti‐GPI IgG‐SC expansion and antibody affinity maturation between 4 and 10 weeks old, followed by slow IgG‐SC response improvement until 35 weeks of age before plateauing and declining. At the peak of the anti‐GPI IgG‐SC response, approximately 50% of IgG‐SC in the PLN, approximately 35% of IgG‐SC in the spleen and bone marrow, and approximately 45% of circulating IgG were specific for GPI. Because anti‐GPI autoantibodies are heavily skewed toward the IgG1 isotype,^6^, ^7^ this fraction may be even higher among IgG1‐SC. The secretion rate of anti‐GPI IgG‐SC remained nevertheless constant in the three anatomic locations over 87 weeks, with peaks of single‐cell secretion rates correlating with the early and rapid IgG‐SC expansion phase.

Using the DropMap droplet microfluidic approach,^13^ we identified herein IgG‐SC without the use of any surface marker, but only on their capability to secrete IgG and among them anti‐GPI IgG. Flow cytometry data on permeabilized CD138^hi^ plasmablasts and plasma cells confirmed the dynamics of anti‐GPI IgG‐SC that we identified through DropMap in the PLN, spleen, and bone marrow. A rapid expansion phase of IgG‐SC correlated with that of KRN TCR‐expressing Tfh cells and of GC B cells, confirming earlier observation on the primordial role of Tfh cells for B cell maturation into plasmablasts and plasma cells.^9^ Plasmablasts have been identified as major cellular players in the K/BxN model as anti‐CD20 B cell depletion experiments, which target plasmablasts but not long‐lived plasma cells, reduced circulating anti‐GPI IgG titers 10‐fold over an eight‐week treatment.^12^ GPI‐specific IgG‐secreting plasmablasts and plasma cells accounted for a large fraction of total IgG‐SC, with a minimum of 20% after 20 weeks in spleen, PLN, and bone marrow, even at later time points. These results indicate a chronic generation of autoreactive B cells throughout the lifetime of K/BxN mice.

Anti‐GPI IgG titers and IgG‐SC numbers did not evolve linearly in K/BxN mice. As reported earlier,^6^ anti‐GPI antibody titers increased rapidly after 3 weeks old, plateaued and slowly decreased. Anti‐GPI IgG‐SC followed the same kinetics with appearance in spleen, PLN, and bone marrow at 3 to 4 weeks old, peaking between 6 and 12 weeks old, and decreasing in numbers thereafter. The median affinities, however, did not vary in any of the three anatomic compartments, supporting the very large predominance of low‐affinity autoreactive IgG‐SC (probably essentially plasmablasts) in this spontaneous RA model. Analyzing data per age group (6–12 weeks old, 23–35 weeks old, and 54–87 weeks old), we found significant temporal decline of the affinities of IgG secreted by anti‐GPI IgG‐SC in the PLN and spleen and, to a lesser extent, in the bone marrow. The highest proportion of anti‐GPI IgG‐SC were identified in draining lymph nodes, although their largest reservoir was the spleen due to organ size differences. The affinity for GPI of the IgG secreted by these IgG‐SC also evolved similarly, with detectable high‐affinity (K_D_ < 1 nM) antibodies only between 6 and 11 weeks old restricted to spleen and PLN. The bone marrow appeared unable to host high‐affinity IgG‐SC (at least detectable numbers thereof), which might be linked to the control of long‐lived plasma cell survival by Foxp3^+^ regulatory T cells in this model.^14^ This peak of medium‐ and high‐affinity IgG‐SC correlates with joint inflammation, which reduces in this model after 10 weeks to be followed by cartilage destruction and bone erosion, indicative of a modification of the immune response. The fact that serum collected during the peak of high‐affinity IgG‐SC was significantly more proarthritic than serum collected at late time points may be a consequence of this affinity maturation burst.

Our study is limited by its focus on affinity measurement throughput. A DropMap assay captures only a small fraction of the IgG‐SC within a given organ. Indeed, one acquisition corresponds to an average of 60,000 to 80,000 droplets, among which only 20,000 contain one or two cells and is thus taken into consideration in our analyses. Assuming the spleen of 3‐week‐old mice contains roughly 30 × 10^6^ cells, we estimate that we screened only 0.07% of the spleen per DropMap. In addition, the in‐droplet assay was designed to capture κ‐light chain antibodies onto the beadline, making it impossible to detect any λ‐light chain anti‐GPI autoantibodies. As mice produce 95% of κ‐light chain antibodies, we nevertheless assume that only rare autoreactive IgG‐SC might have been overseen in our work. Finally, the detection limit of anti‐GPI IgG affinity being 100 nM in DropMap assays, lower‐affinity interactions were not considered, suggesting that the proportion of low‐affinity anti‐GPI IgG‐SC may be even higher than we report herein.

This study provides a direct ex vivo characterization of autoreactive IgG‐SC during initiation and development of arthritis in K/BxN mice. Our results report on the dynamic nature of the autoimmune B cell response in this model that highlights—within a restricted time window—an escape from tolerance leading to antibody affinity maturation.

## AUTHOR CONTRIBUTIONS

All authors contributed to at least one of the following manuscript preparation roles: conceptualization AND/OR methodology, software, investigation, formal analysis, data curation, visualization, and validation AND drafting or reviewing/editing the final draft. As corresponding author, Dr Bruhns confirms that all authors have provided the final approval of the version to be published and takes responsibility for the affirmations regarding article submission (eg, not under consideration by another journal), the integrity of the data presented, and the statements regarding compliance with institutional review board/Declaration of Helsinki requirements.

## Supporting information


**Disclosure form**.

**Appendix S1:** Supplementary Information.
