# Supplementary material for: Lifelong Evolution of Autoreactive Plasma Cell Numbers, Affinity, and Anatomic Location in Arthritic K/BxN Mice
Source: Arthritis Rheumatol. 2026 Feb 17;78(4):821–9. doi: 10.1002/art.43436 (PMC13054446; doi:10.1002/art.43436)
Supplement: Supplementary file 2 — Appendix S1: Supplementary Information. [file ART-78-821-s002.docx]

**SUPPLEMENTAL MATERIAL**

- Supplemental Figures 1-6

**
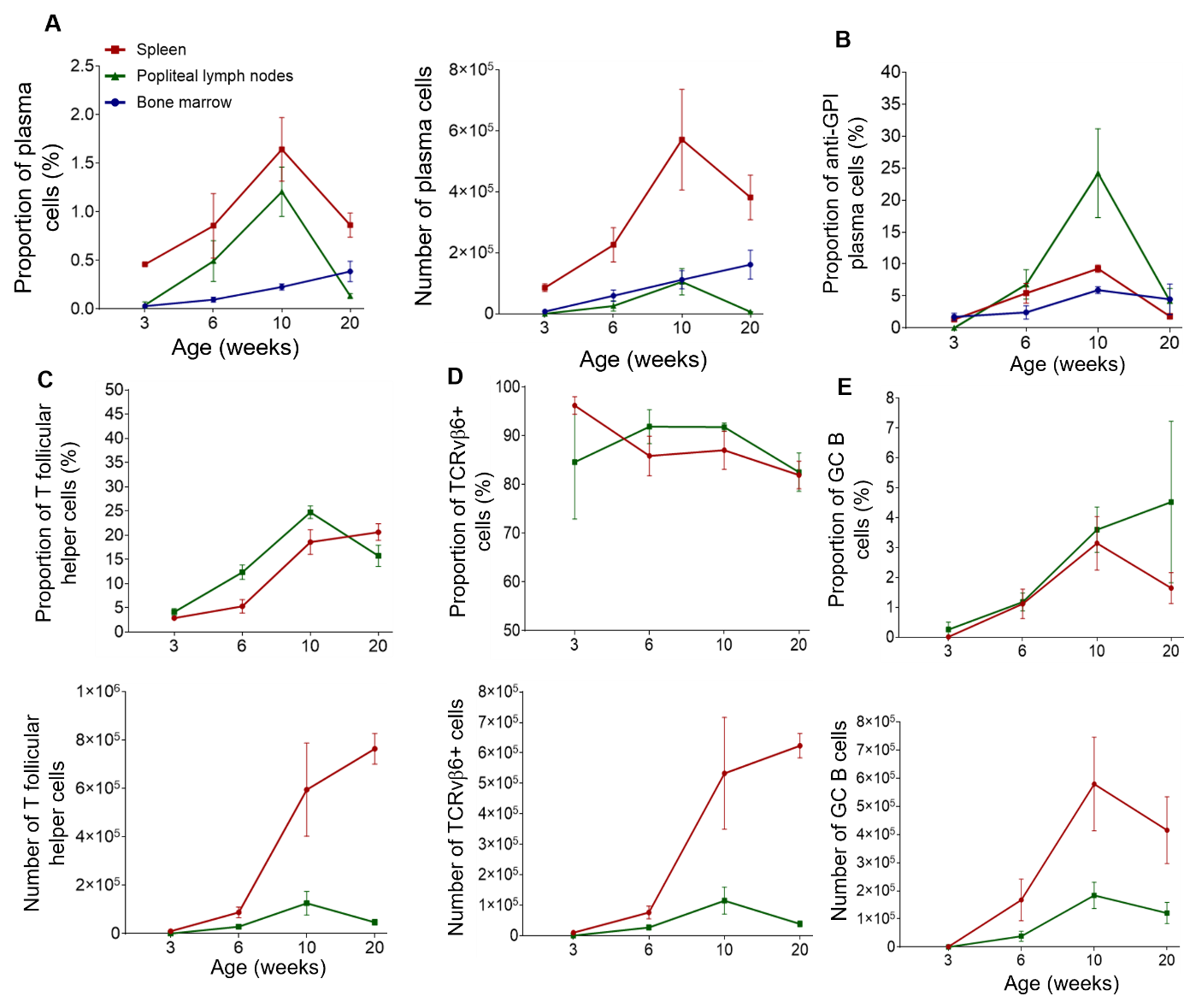
**

**Supplemental Fig. 1. Immune cell dynamics in K/BxN mice from 3 to 20 weeks.** Kinetic evolution in indicated tissues by flow cytometry of the proportion and number of (**A**) total or (**B**) GPI-specific B220^lo/-^ CD138^hi^ plasma cells/plasmablasts (refer to data presented in Fig. 1E). (**C**-**E**) Kinetic evolution in indicated tissues by flow cytometry of the proportion and number of (**C**) total B220^-^ CD4^+^ PD-1^hi^ CXCR5^hi^ Tfh cells, (**D**) TCR^vβ6+^ Tfh cells, (**E**) germinal center B220^+^ CD4^-^ GL7^+^ FAS^+^ B cells. Cells were gated from alive single cells. Data represents the mean of six mice at week 3, and the mean of three mice at 6, 10 and 20 weeks. (**A**-**E**) Data is represented as mean ± SEM.


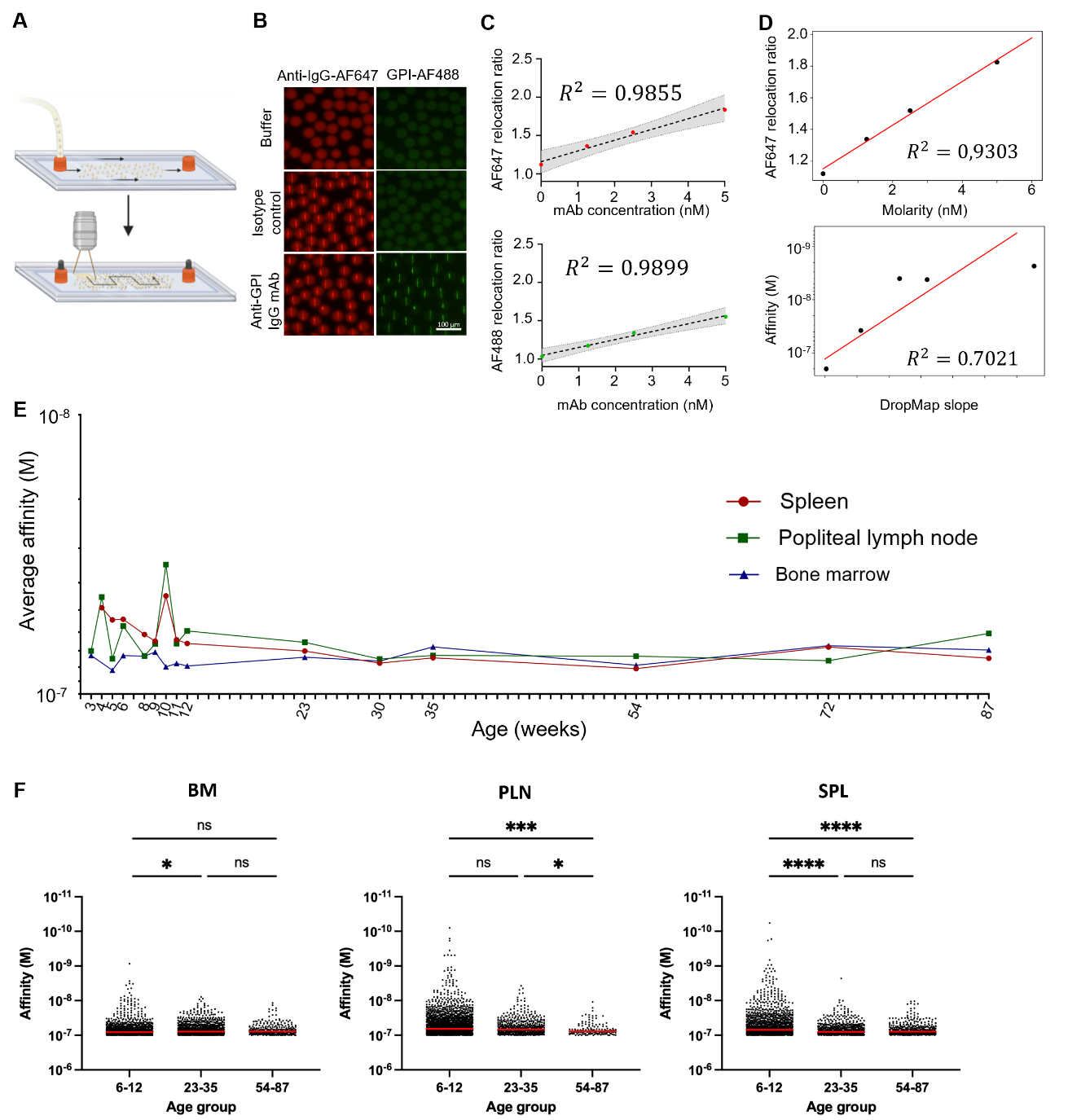


**Supplemental Fig. 2.** (**A**) (Top) Schematic of the DropMap observation chamber filling with droplets, with the oil flow represented by arrows. (Bottom) Schematic of image acquisition of the observation chamber containing immobilized droplets. The microscope objective scans the chamber and generates a 12×12 matrix of 144 individual fields. (**B**) Example of droplets containing no mAb (Buffer), 5 nM mouse IgG isotype control (Isotype control), or 5 nM anti-GPI IgG mAb clone D24.4 (Anti-GPI IgG mAb), showing fluorescence relocation of anti-IgG F(ab′)_2_–Alexa Fluor 647 (Anti-IgG-AF647) and of GPI-Alexa Fluor 488 (GPI-AF488); scale bars: 100 μm. (**C**) Measurements of fluorescence relocation of encapsulated anti-GPI mAb clone D24.4 at 0, 1.25, 2.5 and 5 nM. Linear regression was performed, and 95% confidence intervals are depicted in gray. (**D**) Reference curves calibrated with four anti-GPI IgG Abs with known affinity for GPI and one isotype control. Each dot represents the values obtained for one anti-GPI mAb or the isotype control. (Top) Secretion rate reference curve; (bottom) anti-GPI affinity reference curve. Linear regression was performed, and R^2^ values are indicated. **(E)** Kinetic evolution of anti-GPI ASCs affinity in indicated tissues. (**F**) Kruskal-Wallis test with Dunn’s test for multiple comparisons on aged-grouped affinity values from DropMap data from Figure 2C-E in bone marrow (BM), popliteal lymph nodes (PLN) and spleen (SPL). *: p<0.05, ***: p<0.001, ****: p<0.0001.


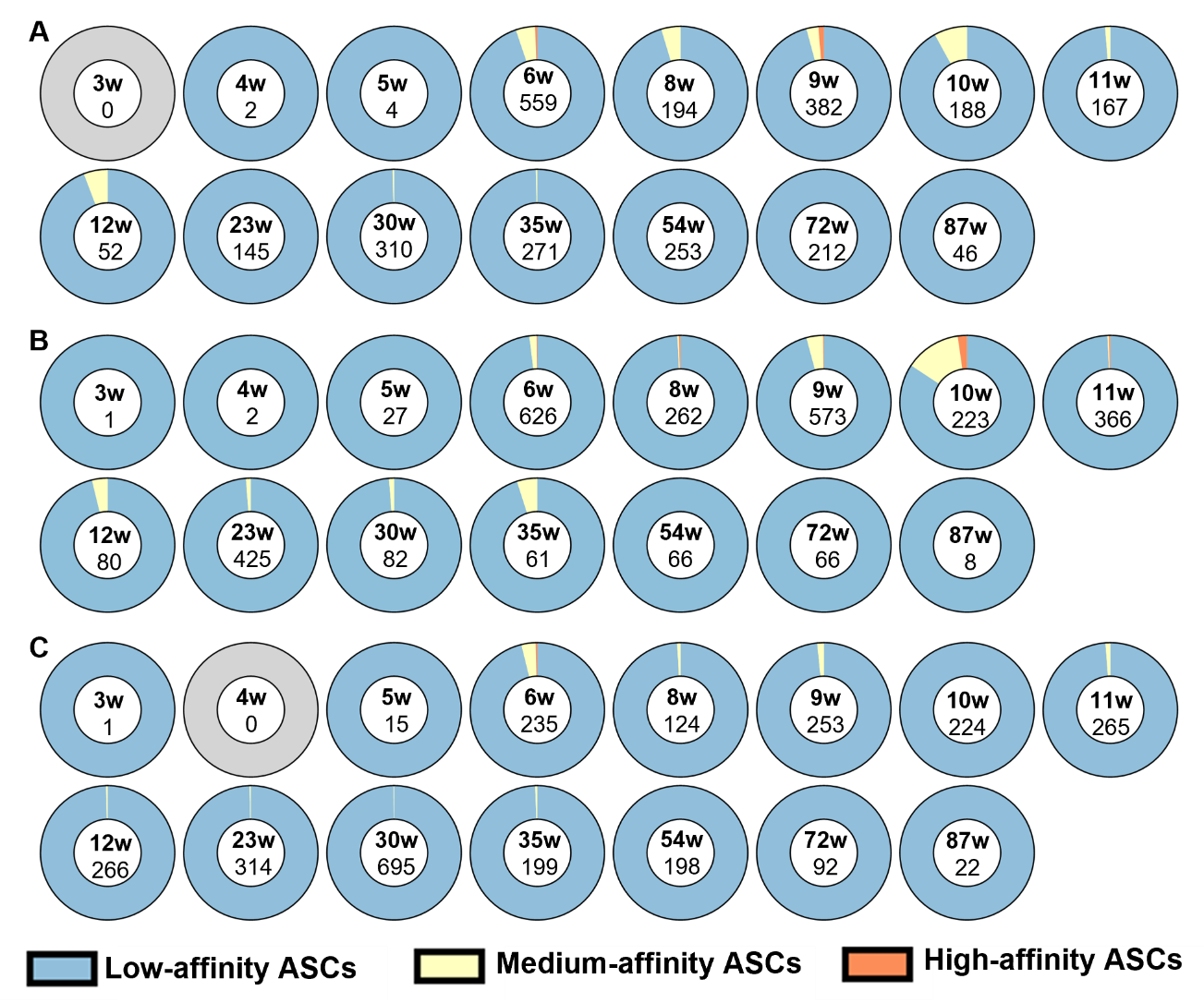


**Supplemental Fig. 3.** Proportions of low (blue), medium (yellow) and high affinity (orange) GPI-specific IgG-SCs for all mice analyzed in Figure 2C-E for each time point for (**A**) spleen, (**B**) popliteal lymph nodes and (**C**) bone marrow. The time point in weeks (w) and the total GPI-specific IgG-SCs detected at that time point for all mice analyzed are indicated in the center of the donut.

**
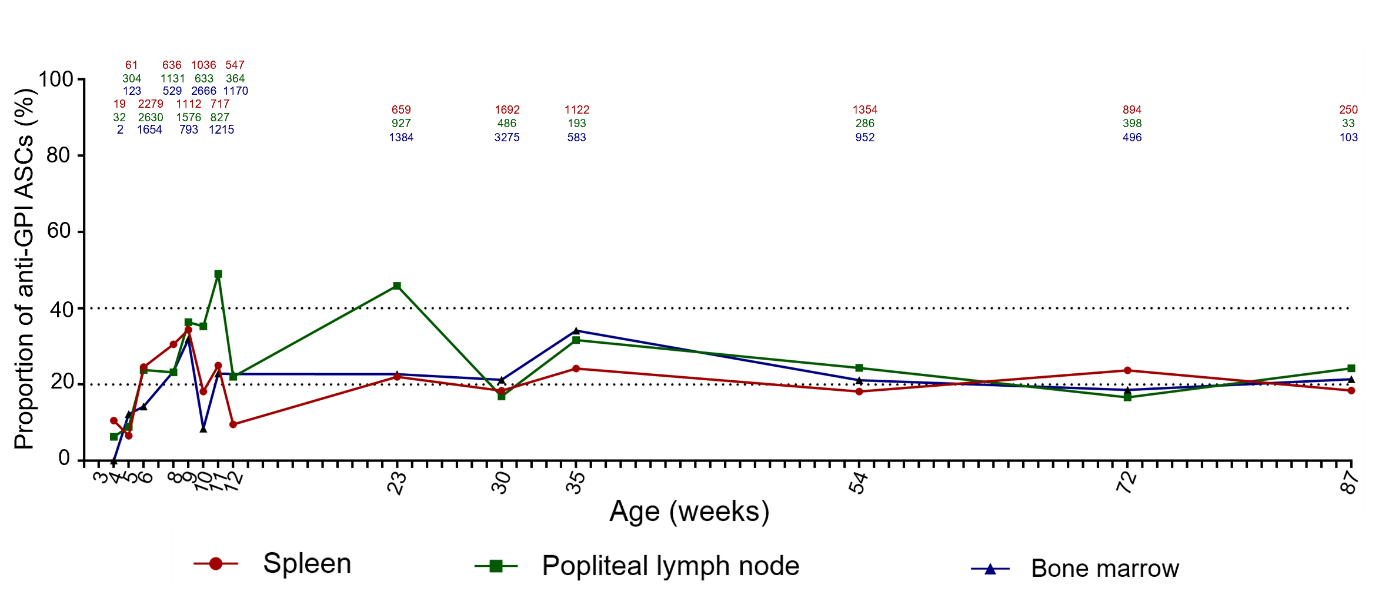
**

**Supplemental Fig. 4.** Proportions of GPI-specific IgG-SCs among all IgG-SCs in spleen, popliteal lymph nodes and bone marrow over time in spleen (red), popliteal lymph nodes (green) and bone marrow (blue). The total number of IgG-SCs detected for each organ (red, spleen; green, popliteal lymph nodes; blue, bone marrow) and each timepoint are indicated at the top of the graph


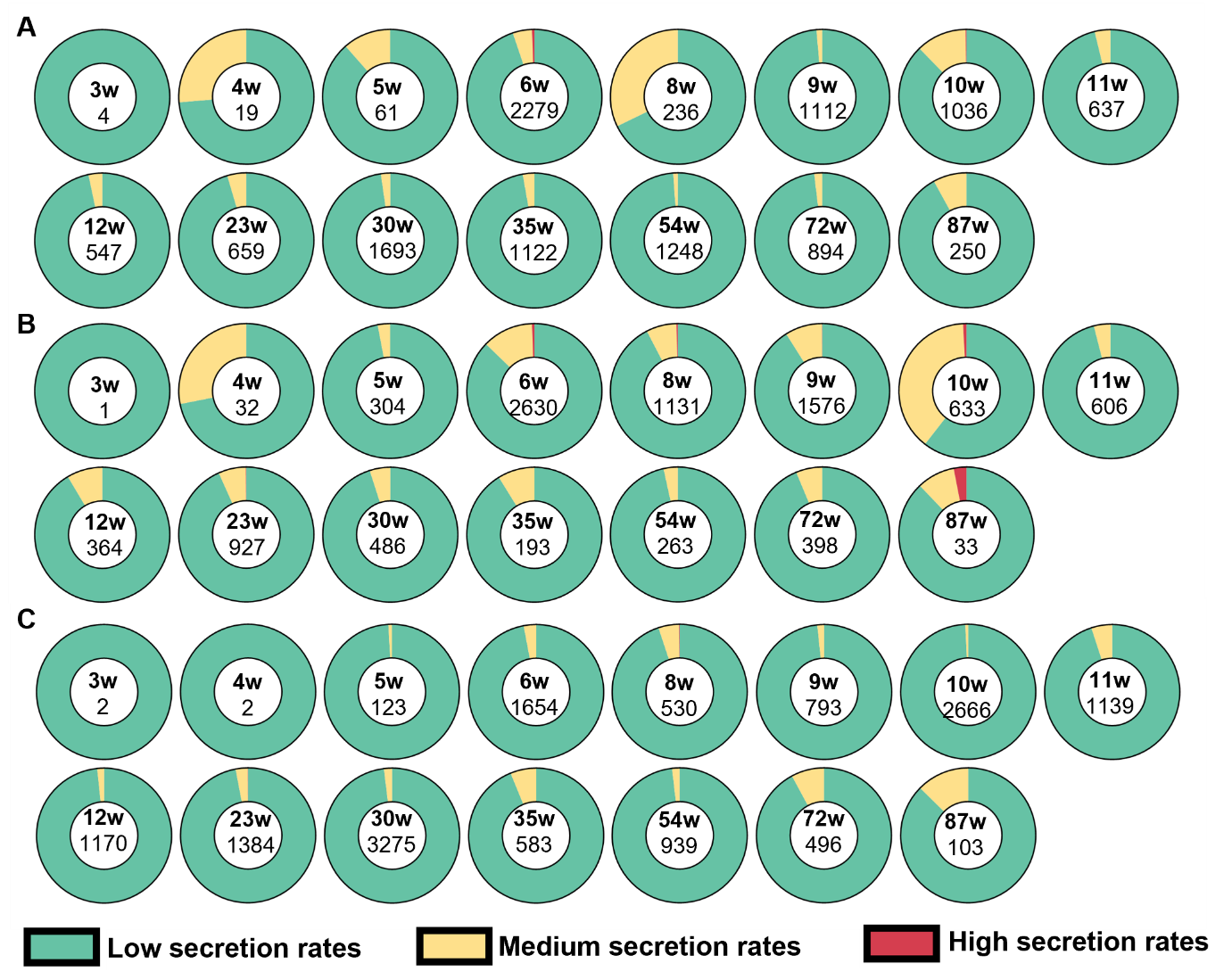


**Supplemental Fig. 5.** Proportions of low (blue), medium (yellow) and high (orange) IgG secreters among IgG-SCs for all mice analyzed in Figure 3A-C for each time point for (**A**) spleen, (**B**) popliteal lymph nodes and (**C**) bone marrow. The time point in weeks (w) and the total IgG-SCs detected at that time point for all mice analyzed are indicated in the center of the donut.


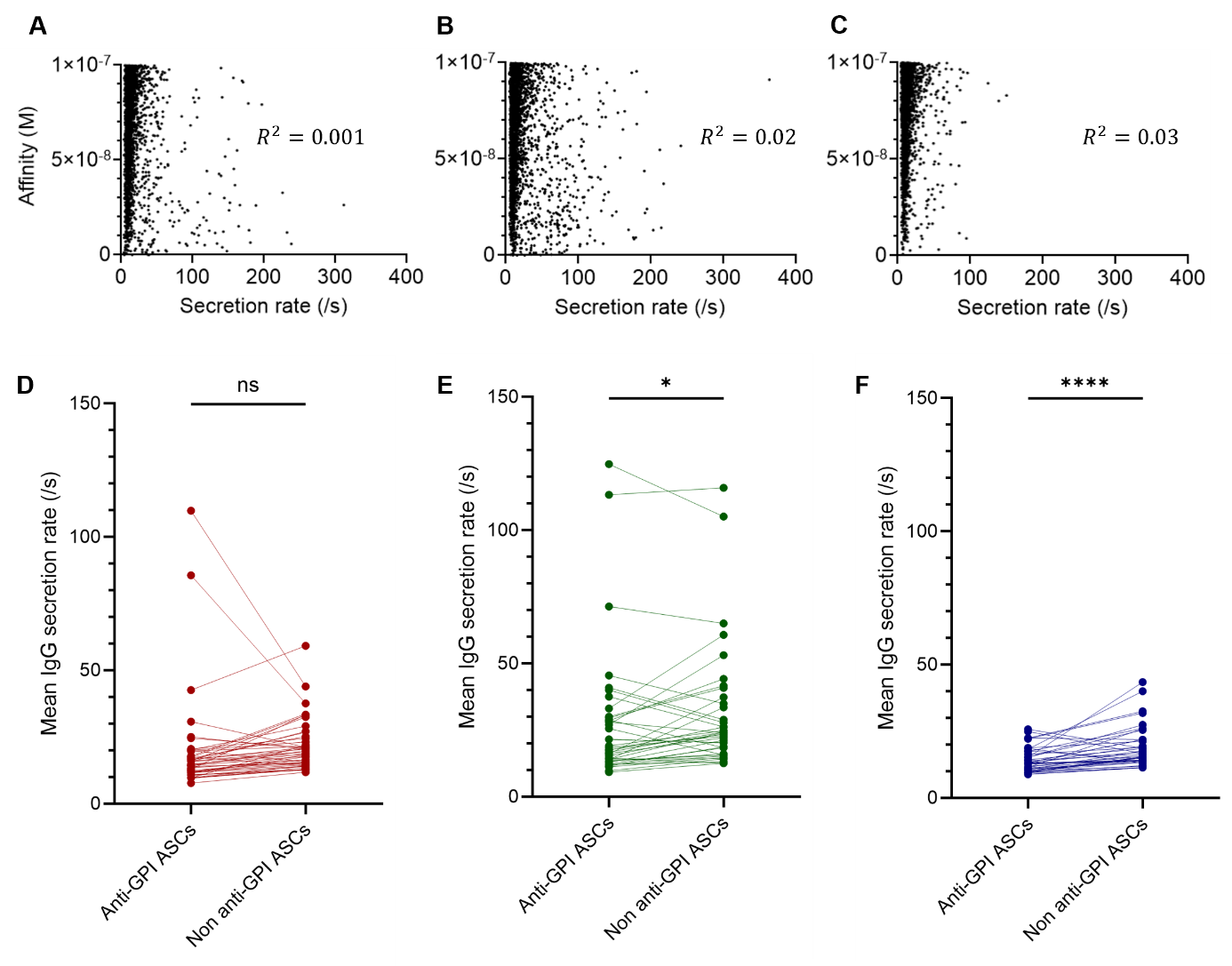


**Supplemental Fig. 6.** (**A**-**C**) Absence of correlation between affinity for GPI and secretion rate of IgG-SCs detected in (**A**) spleen, (**B**) popliteal lymph nodes and (**C**) bone marrow compiled from all K/BxN mice analyzed from 3 to 87 weeks. R^2^ are indicated. (**D**-**F**) Comparison of mean secretion rates of anti-GPI IgG-SCs and non-specific IgG-SCs of K/BxN mice from 3 to 87 weeks of age analyzed in this study. Each dot represents the mean secretion rate of all IgG-SCs detected for one mouse restricted to samples containing at least 10 autoreactive and 10 non-autoreactive ASCs for (**A**) spleen, (**B**) popliteal lymph nodes and (**C**) bone marrow. (**D**-**F**) Data were compared using a paired t-test. ns: not significant, *: p<0.05, ****: p<0.0001.
